# Left ventricular mass and myocardial scarring in women with hypertensive disorders of pregnancy

**DOI:** 10.1136/openhrt-2020-001273

**Published:** 2020-08-06

**Authors:** Odayme Quesada, Ki Park, Janet Wei, Eileen Handberg, Chrisandra Shufelt, Margo Minissian, Galen Cook-Wiens, Parham Zarrini, Christine Pacheco, Balaji Tamarappoo, Louise E J Thomson, Daniel S Berman, Carl J Pepine, Noel Bairey Merz

**Affiliations:** 1 Smidt Heart Institute, Cedars-Sinai Medical Center, Los Angeles, California, USA; 2 Barbra Streisand Women's Heart Center, Cedars-Sinai Medical Center, Los Angeles, California, USA; 3 Cardiovascular Medicine, University of Florida College of Medicine, Gainesville, Florida, USA; 4 Biostatistics and Bioinformatics Research Center, Cedars-Sinai Medical Center, Los Angeles, California, USA; 5 S Mark Taper Foundation Imaging Center, Cedars-Sinai Medical Center, Los Angeles, California, USA

**Keywords:** risk factors, coronary artery disease, hypertension, MRI

## Abstract

**Aims:**

Hypertensive disorders of pregnancy (HDP) predict future cardiovascular events. We aim to investigate relations between HDP history and subsequent hypertension (HTN), myocardial structure and function, and late gadolinium enhancement (LGE) scar.

**Methods and results:**

We evaluated a prospective cohort of women with suspected ischaemia with no obstructive coronary artery disease (INOCA) who underwent stress/rest cardiac magnetic resonance imaging (cMRI) with LGE in the Women’s Ischemia Syndrome Evaluation-Coronary Vascular Dysfunction study. Self-reported history of pregnancy and HDP (gestational HTN, pre-eclampsia, toxaemia and eclampsia) were collected at enrollment. In our cohort of 346, 20% of women report a history of HDP. HDP history was associated with 3.2-fold increased odds of HTN. Women with a history of *both* HDP and HTN had higher cMRI measured left ventricular (LV) mass compared with women with HDP only (99.4±2.6 g vs 87.7±3.2 g, p=0.02). While we found a similar frequency of LGE scar, we observed a trend towards increased LGE scar size (5.1±3.4 g vs 8.0±3.4 g, p=0.09) among the women with HDP history compared to women without.

**Conclusion:**

In a high-risk cohort of women with suspected INOCA, 20% had a history of HDP. Women with HDP history were more likely to develop HTN. Our study demonstrates higher LV mass in women with HDP and concomitant HTN. Although the presence of LGE scar was not different in women with and without HDP history, we observed a trend towards larger scar size in women with HDP. Future studies are needed to better assess the relationship of HDP and cardiac morphology and LGE scarring in a larger cohort of women.

**Key questions**

**What is already known about this subject?**

- Hypertensive disorders of pregnancy (HDP) are associated with increased risk of cardiovascular disease and mortality.

**What does this study add?**

- Our study demonstrates higher left ventricular mass in women with HDP and concomitant hypertension (HTN) history and a trend towards larger LGE myocardial scar size in women with HDP. Future studies are needed to better assess the relationship of HDP and left ventricular morphology and myocardial scarring in a larger cohort of women.

**How might this impact on clinical practice?**

- Our findings support HTN surveillance in women with HDP who may be at higher risk for abnormalities in cardiac morphology.

## Introduction

Increasing evidence has led to a wider recognition of women-specific risk factors for cardiovascular disease (CVD). These include hypertensive disorders of pregnancy (HDP), such as gestational hypertension (HTN) and pre-eclampsia, which combined complicate up to 10% of pregnancies and are characterised by de novo HTN after 20 weeks gestation.^1–3^ Although the pathophysiology is poorly understood, large cohort studies have found that pre-eclampsia is associated with up to an eightfold higher risk of CVD and mortality compared with women with healthy normotensive pregnancies.^4–8^ These findings emphasise the importance of understanding women-specific risk factors for CVD.

Cardiac magnetic resonance imaging (cMRI) with late gadolinium enhancement (LGE) can be used to evaluate ventricular morphology and function, detect myocardial scar and quantify scar size with high accuracy.^9 10^ cMRI measures, particularly increased left ventricular (LV) mass, presence of ischaemic and non-ischaemic scar as determined by LGE imaging are independent risk factors for major adverse cardiovascular events.^11–16^

Research on the associations between HDP and CVD is limited. Therefore, we investigated the risk of developing HTN decades after the index pregnancy complicated by HDP and relationship of history of HDP and HTN with cMRI measured LV morphology and function, and presence and size of LGE myocardial scar in women with ischaemia with no obstructive coronary artery disease (INOCA) in the Women’s Ischemia Syndrome Evaluation-Coronary Vascular Dysfunction (WISE-CVD) cohort.^17^ We hypothesise that women with HDP history will have abnormalities in LV morphology and function and more likely to have LGE myocardial scar.

## Methods

### Study population

This investigation was part of the National Heart, Lung, and Blood Institute-sponsored prospective multicentre WISE-CVD study (URL: http://www.clinicaltrials.gov, unique identifier: NCT00832702). WISE-CVD was a prospective study of women with suspected sign and symptoms of INOCA (defined as <50% luminal diameter in any major coronary artery) on invasive angiography.^18^ Subjects were recruited from January 2009 to August 2015 at Cedars-Sinai Medical Center, Los Angeles, California or University of Florida, Gainesville, Florida. The protocol was approved by the Institutional Review Board at each site and all participants provided written informed consent.

As previously described women with signs and symptoms of ischaemia undergoing clinically indicated coronary angiography, age ≥21 years and competent to give informed consent were included. Exclusion criteria included acute coronary syndrome (defined by the American College of Cardiology/American Heart Associationcriteria),^19^ acute myocardial infarction; concurrent cardiogenic shock or inotropic or intra-aortic balloon support; prior or planned percutaneous coronary intervention or coronary artery bypass graft (CABG); primary valvular heart disease clearly indicating need for valve repair or replacement; chest pain with a non-ischaemic aetiology (eg, pericarditis, pneumonia, oesophageal spasm); conditions that preclude accurate or safe testing, or prognostic follow-up, specifically contraindications to cMRI (eg, implantable cardioverter defibrillator, pacemaker, untreatable claustrophobia or known angio-oedema, severe renal impairment (estimated glomerular filtration rate (eGFR) <45 mL/min); prior non-cardiac illness with an estimated life expectancy <4 years; and obstructive coronary artery disease defined as ≥50% luminal diameter stenosis in ≥1 epicardial coronary artery, assessed visually at the time of angiography.^18^

Self-reported history of pregnancy, HDP and HTN were collected at time of enrollment. Questionnaires specifically collected data on history of gestational HTN, pre-eclampsia, toxaemia and eclampsia. HTN was collected based on patient-reported medical history of HTN. Among the 374 women who completed cMRI in the WISE-CVD study, we included 346 women who reported history of at least one pregnancy and completed questions on history of HDP in our analysis.

### Patient and public involvement

It was not appropriate or possible to involve patients or the public in the design, or conduct, or reporting or dissemination plans of our research.

### cMRI protocol and analyses

Stress and rest cMRI were performed at time of enrollment on a 1.5-Tesla MR scanner (Magnetom Avanto, Siemens Healthcare Erlangen, Germany) in the supine position with ECG gating. All subjects were asked to hold all their cardiac medications 24–48 hours prior to cMRI. A highly standardised protocol was used and included assessment of ventricular function and morphology and LGE imaging.^18^ In brief, LGE images were acquired in 10–12 short axis slices, one horizontal long axis slice and one vertical long axis slice in the same positions as the LV function cine images. A single shot trufi-based sequence was used with heart rate-based temporal resolution and echo time minimised at 0.98 ms. A ‘TI scout’ image was obtained followed by single shot inversion recovery TrueFISP images 10 min after last gadolinium injection.

The WISE-cMRI core lab analysed LV function and morphology using commercially available software (CAAS MRV V.3.4, PIE Medical Imaging) as previously described.^20–22^ Epicardial and endocardial borders of short-axis cine images were manually traced and postprocessing software was used to generate volume–time curves used for LV volumes and LV mass. Stroke volume was calculated as end-diastolic volume minus end-systolic, and ejection fraction as stroke volume divided by end-diastolic volume. LGE quantification was performed by a single experienced operator using associated postprocessing software (QMass, Medis) by defining endocardial and epicardial borders using the short-axis images and the full width at half-maximum method. LGE myocardial scar pattern was evaluated visually and defined as atypical scar pattern when mid-myocardial or epicardial scar pattern was present; and typical scar pattern when scar pattern was subendocardial or transmural and localised to a coronary artery distribution as previously described.^23^

### Statistical analyses

The 346 women with a pregnancy history were divided into two groups: those with HDP and those without. Variables were summarised using mean and SD, or frequency and per cent if categorical, median and IQR was used to report LGE myocardial scar size. Baseline clinical and demographic variables were tested between these two groups using Pearson χ^2^ tests, or Fisher’s exact test for categorical variables that had low expected cell counts, or t-tests for continuous variables, unless non-normal distributions were present where a Wilcoxon rank-sum test was used.

The primary outcome assessed was HTN and secondary outcome was LV mass. A logistic regression model was used with history of HTN as the outcome, and groups with and without history of HDP as the explanatory factors, adjusting for age, body mass index (BMI), income category, diabetes and number of pregnancies. Additionally, linear regression models were used to examine the association of history of HDP and HTN with LV mass adjusting for history of HDP, history of HTN and interaction between the two. Additionally, adjusted multiple linear regression models accounting for age, BMI and systolic blood pressure (SBP) were performed. Tukey post hoc adjustment was used for pair-wise comparisons. All hypothesis tests used a significance level of 0.05. All analyses were done in SAS V.9.4 (SAS Institute, Cary, North Carolina, USA).

## Results

Overall, 20% (68/346) of the women included in this analysis report history of at least one HDP. Pertinent demographics and clinical characteristics are summarised in table 1. Women with HDP history were younger (p=0.05) and had a higher mean BMI (p<0.001) compared with those without. There were no differences in coronary severity score and per cent of coronary artery stenosis on invasive coronary angiography between the group with and without HDP history. History of HDP was associated with 3.2-fold increased odds of HTN compared with women without HDP after adjusting for age, BMI, history of diabetes, number of pregnancies and income level (95% CI 1.4 to 7.3, adjusted p=0.005). cMRI LV structure and function parameters were similar between both groups (table 2).

**Table 1:** Demographics and clinical characteristics of women with INOCA with and without history of HDP

Characteristics	History of a HDP		P value
	No (n=278)	Yes (n=68)	
Demographics			
Age, years	55.6±10.6	53.1±9.6	**0.05**
BMI, kg/m^2^	27.4±6.6	31.7±7.5	**<0.001**
Race/ethnicity			0.7
White/Non-Hispanic	207 (74.5%)	52 (76.5%)	
Black/African–American	20 (7.2%)	7 (10.3%)	
Hispanic/Latin	24 (8.6%)	4 (5.9%)	
Annual income			**0.04**
US$0–US$49 000	84 (31.2%)	32 (48.5%)	
US$50 000–US$99 000	71 (26.4%)	12 (18.2%)	
US$100 000+	114 (42.4%)	22 (33.3%)	
Clinical characteristics			
HTN	92 (35.3%)	41 (65.1%)	0.002*
Dyslipidaemia	42 (18.9%)	11 (22%)	0.7
Diabetes mellitus	30 (11%)	12 (18.2%)	0.1
Ever smoker	115 (41.4%)	25 (37.3%)	0.6
Cardiovascular medications			
ACE-I or ARB	65 (24.6%)	22 (32.8%)	0.2
Beta blocker	83 (31.1%)	27 (40.3%)	0.2
Calcium channel blocker	58 (21.9%)	19 (28.8%)	0.3
Diuretic	34 (12.7%)	14 (20.9%)	0.1
Postmenopausal	202 (72.7%)	52 (76.5%)	0.6
Number of pregnancies	3±2, 3	4±1, 4	**0.01**
Coronary severity score	9.2±4.2, 8.5	10.3±4.5, 9.6	0.1
Angiographic findings			0.8
No CAD	12 (6.4%)	4 (8.3%)	
No obstructive CAD	164 (87.2%)	40 (83.3%)	

Values are N (%), mean±SD, median (range).

No CAD defined as <20% coronary artery stenosis, no obstructive CAD defined as 20%–50% coronary artery stenosis.

Bold indicates significant p value </= 0.05

*Adjusted for age, BMI, diabetes, annual income, number of pregnancies.

ACE-I, angiotensin-converting enzyme inhibitors; ARB, angiotensin receptor blockers; BMI, body mass index; CAD, coronary artery disease; HDP, hypertensive disorders of pregnancy; HTN, hypertension; INOCA, ischaemia with no obstructive coronary artery disease.

**Table 2:** cMRI haemodynamics, LV morphology and function in women with INOCA with and without history of HDP

cMRI variables	History of a HDP		P value
	No (n=278)	Yes (n=68)	
Heart rate (bpm)	68.7±10.6	68.5±12.4	0.9
SBP (mm Hg)	129.7±20.8	134.4±18.7	0.1
DBP (mm Hg)	62.7±13.4	65.7±12.3	0.1
Ejection fraction (%)	68.2±6.8	68.3±7.6	0.6*
End-diastolic volume (mL)	121.1±23.8	128.7±24.1	0.5*
End-systolic volume (mL)	38.9±13.23	41.3±13.3	0.1
Stroke volume (mL)	82.0±16.5	86.3±17.6	0.7*
Mass to volume ratio	0.8±0.1	0.8±0.2	0.6
LGE %	20 (7.2%)	3 (4.4%)	0.6

Values are mean±SD, or median (range).

*Adjusted for age, BMI.

BMI, body mass index; cMRI, cardiac MRI; DBP, diastolic blood pressure; HDP, hypertensive disorders of pregnancy; INOCA, ischaemia with no obstructive coronary artery disease; LGE, late gadolinium enhancement; LV, left ventricular; SBP, systolic blood pressure.

**Figure 1:**
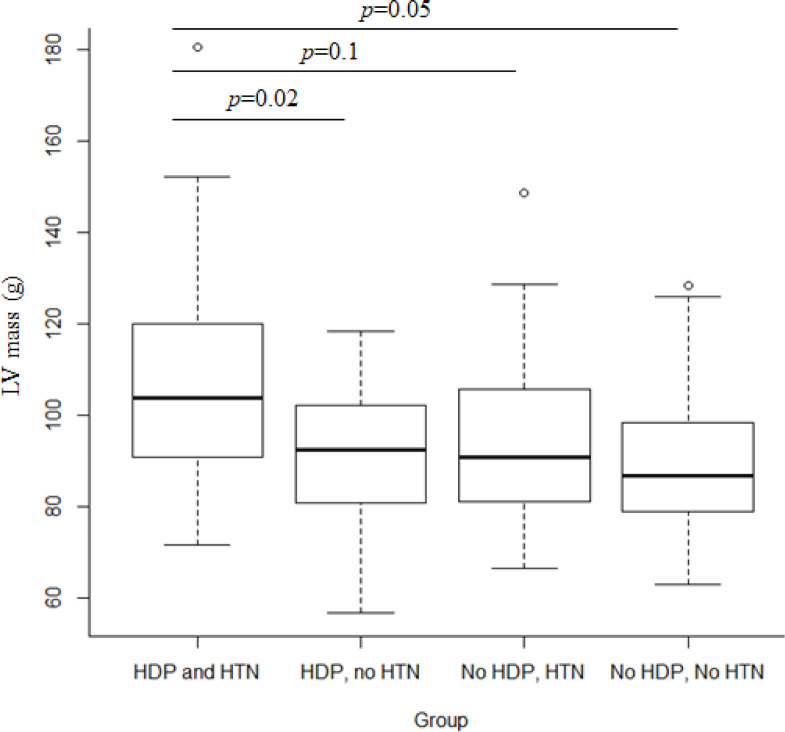
Box plot of LV mass in women with history of HDP and HTN box plot showing that women with a history of both HDP and HTN have the highest average LV mass to women with HDP only (HDP, no HTN), HTN only (no HDP, HTN) or neither (no HDP, no HTN). HDP, hypertensive disorders of pregnancy; HTN, hypertension; LV, left ventricular.

In unadjusted and adjusted models, we found an interaction between history of HDP and HTN in relation to cMRI-measured LV mass (interaction adjusted p=0.01). Women with a history of both HDP and HTN had the highest average LV mass compared with women with HDP only (HDP, no HTN), HTN only (no HDP, HTN) or neither (no HDP, no HTN, figure 1). The adjusted model showed that LV mass increased as BMI increased (by 1.3 per unit BMI, p<0.001). Women with a history of *both* HDP and HTN had a significantly higher LV mass compared with women with HDP only (99.4±2.6 g vs 87.7±3.2 g, p=0.02) and women with neither (99.4±2.6 g vs 91.9±1.2 g, p=0.05) after adjusting for age, BMI and SBP.

**Figure 2:**
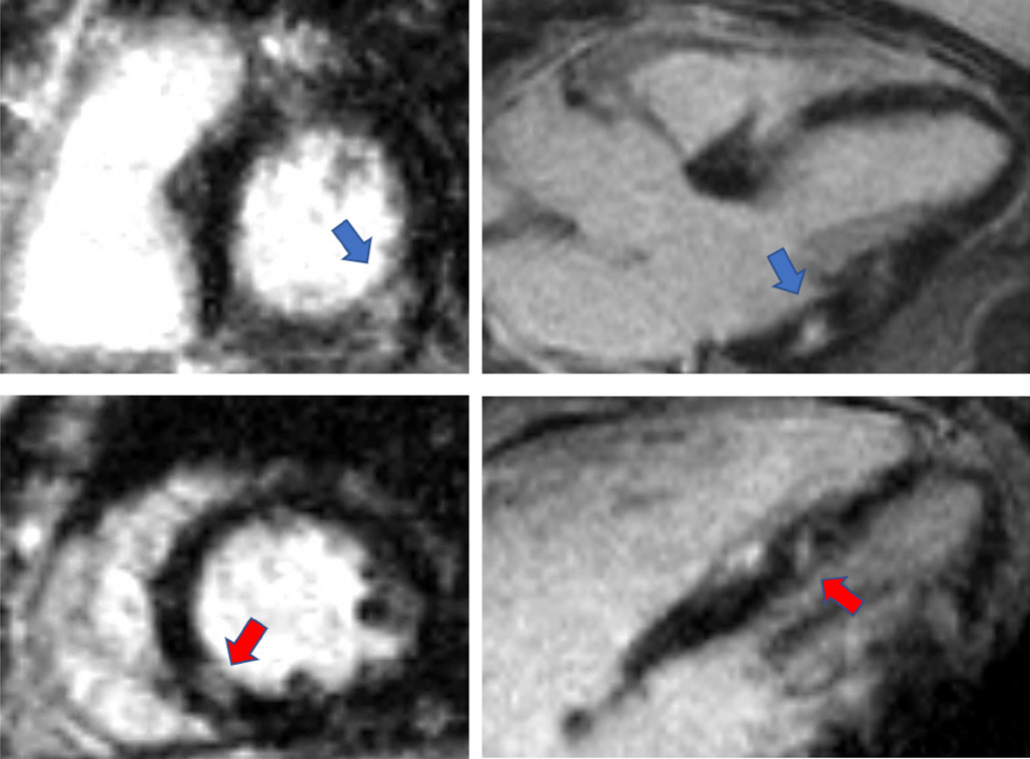
Myocardial scar in woman with ischaemia with no obstructive CAD and HDP sample case of a participant with INOCA and history of HDP disorder of pregnancy showing inferolateral subepicardial LGE myocardial scar (blue arrow) and inferoseptal subendocardial LGE myocardial scar (red arrow). CAD, coronary artery disease; HDP, hypertensive disorders of pregnancy; INOCA, ischaemia with no obstructive coronary artery disease; LGE, late gadolinium enhancement.

While there was a relatively similar frequency of cMRI LGE myocardial scar, we observed a trend towards increased LGE myocardial scar size (5.1±3.4 g, 4.0 (1.9, 18.0) vs 8.0±3.4 g, 8.3 (4.5, 11.2), p=0.09) in women with history of HDP compared with women withouttable 2. Among the women with history of HDP with LGE myocardial scar, 67% had typical scar pattern (vascular pattern consistent with coronary distribution) and 33% had atypical scar pattern (non-vascular pattern). Among women without history of HDP, 78% had typical pattern and 22% had atypical pattern. Sample case of a participant with INOCA and history of HDP showed inferolateral transmural LGE myocardial scar and inferoseptal subendocardial LGE myocardial scar (figure 2).

## Discussion

Studies assessing cardiac structure and function in women with history of HDP are limited, to our knowledge our study is the first to use advanced imaging with LGE to evaluate myocardial scar in a high-risk group of women with history of HDP. In our cohort of women with signs and symptoms of INOCA, 20% had a history of HDP. Our study confirms that women with HDP are more likely to go on to develop HTN. We also found that HDP history is related to cMRI-determined higher LV mass in women with concomitant HTN. Although LGE myocardial scar was not different in women with and without HDP history, we observed a a trend towards larger scar size in women with HDP.

In accordance with prior studies, we find that women with HDP history have 3.2-fold increase risk of developing HTN later in life.^24^ During pregnancies complicated by HDP there is a pronounced increase in LV mass in response to the increased LV workload from the sudden development of HTN that leads to abnormal cardiac remodelling.^25 26^ Unlike healthy pregnancies, in which the physiologic increase in LV mass and dimensions reverses a few weeks post partum, studies have shown that in HDP abnormal cardiac remodelling can persist up to 2 years post partum.^26–28^ However, the extent to which these abnormalities in cardiac remodelling persist decades later remains unclear. We found that the combination of HDP and HTN was associated with higher LV mass similar to Scantlebury *et al* who found that history of HDP was a risk factor for LV hypertrophy mediated by HTN using echocardiography.^28^ Further, Ghossein-Doha *et al* showed that increased LV mass at 10 months post partum was a predictor for the development of HTN in women whose blood pressure had normalised after a pregnancy complicated by HDP.^25^ Our study supports this inter-relationship between HDP, adverse cardiac remodelling and HTN and suggests that both HDP and HTN may lead to adverse cardiac remodelling decades later.

The Danish cohort is the only other cohort that has used cMRI with gadolinium to evaluate for myocardial scar with LGE in otherwise healthy women with history of HDP; however, they did not find evidence of myocardial scar in the 28 women with history of HDP.^29^ In our cohort, 23 had LGE with similar frequency in those with and without history of HDP, this difference is likely due to the fact that our cohort is composed of high-risk women with suspected INOCA. Our trend towards larger LGE myocardial scar in women with history of HDP leads us to hypothesise that HDP history may contribute to irreversible myocardial injury that needs further evaluating in future larger studies. The mechanisms are unknown, and could be via HTN, abnormal cardiac remodelling, myocardial infarction or other comorbidities.

Our study has limitations. Our findings are limited to a high-risk cohort of women with suspected INOCA that is not representative of the general population. History of HDP and HTN were collected through self-report, which is subject to recall bias.^30–33^Although maternal recall of HDP has a high specificity, its modest predictive value is a limitation of our study as it is for most studies on HDP conducted decades after pregnancy period.^30 33^ We were unable to account for presence of HTN prior to pregnancy or duration of HTN postpregnancy as this data were not collected. Additionally, we were underpower to assess the relationship between HDP and cardiovascular outcomes.

## Conclusions

In summary, in our cohort of women with signs and symptoms of INOCA, 20% had a history of HDP. Our study is consistent with prior work and confirms that women with HDP are more likely to go on to develop HTN. We also found higher LV mass in women with concomitant history of HDP and HTN, supporting the current guidelines on increased HTN surveillance in women with history of HDP. Although the presence of LGE myocardial scar was not different in women with and without HDP history, we observed a trend towards larger LGE myocardial scar size in women with HDP. Future studies are needed to better assess the relationship of HDP and LV morphology and function and LGE myocardial scarring in a larger cohort of women.
